# Supplementary material for: Modelling Skylarks (Alauda arvensis) to Predict Impacts of Changes in Land Management and Policy: Development and Testing of an Agent-Based Model
Source: PLoS One. 2013 Jun 6;8(6):e65803. doi: 10.1371/journal.pone.0065803 (PMC3675089; doi:10.1371/journal.pone.0065803)
Supplement: Supporting Information S4 — The skylark ODdox as a zipped archive. (ZIP) [file pone.0065803.s004.zip › Skylark_ODdox/class_pesticide-members.html]

ALMaSS Skylark ODdox: Member List


ALMaSS Skylark ODdox
 2.0


- Main Page
- Related Pages
- Classes
- Files

- Class List
- Class Index
- Class Hierarchy
- Class Members

Pesticide Member List

This is the complete list of members for Pesticide, including all inherited members.

|  |  |  |
| --- | --- | --- |
| DailyQueueAdd(LE \*a\_element\_sprayed, double a\_amount) | Pesticide |  |
| DailyQueueClear(void) | Pesticide | protected |
| DailyQueueProcess(void) | Pesticide | protected |
| DiffusionFunction(double a\_dist\_meters) | Pesticide | protected |
| DiffusionMaskInit(void) | Pesticide | protected |
| DiffusionMaskInitTest(void) | Pesticide |  |
| DiffusionSprayPixel(int a\_x, int a\_limit\_x, int a\_y, int a\_limit\_y, double a\_amount) | Pesticide | inlineprotected |
| ElementIsWater(int a\_x, int a\_y) | Pesticide | inlineprotected |
| m\_corr\_x | Pesticide | protected |
| m\_corr\_y | Pesticide | protected |
| m\_daily\_spray\_queue | Pesticide | protected |
| m\_diffusion\_mask | Pesticide | protected |
| m\_land | Pesticide | protected |
| m\_map | Pesticide | protected |
| m\_pest\_daily\_decay\_frac | Pesticide | protected |
| m\_pest\_map\_height | Pesticide | protected |
| m\_pest\_map\_main | Pesticide | protected |
| m\_pest\_map\_mask | Pesticide | protected |
| m\_pest\_map\_size | Pesticide | protected |
| m\_pest\_map\_twin | Pesticide | protected |
| m\_pest\_map\_width | Pesticide | protected |
| m\_prop | Pesticide | protected |
| m\_something\_to\_decay | Pesticide | protected |
| m\_x\_excess | Pesticide | protected |
| m\_y\_excess | Pesticide | protected |
| MainMapDecay(void) | Pesticide | protected |
| Pesticide(RasterMap \*a\_land, Landscape \*a\_map) | Pesticide |  |
| SavePPM(double \*a\_map, int a\_beginx, int a\_width, int a\_beginy, int a\_height, char \*a\_filename) | Pesticide |  |
| SupplyPesticide(int a\_x, int a\_y) | Pesticide | inline |
| SupplyPesticide(int a\_polyref) | Pesticide | inline |
| Test(Landscape \*a\_map) | Pesticide |  |
| Tick(void) | Pesticide |  |
| TwinMapAddToMain(void) | Pesticide | protected |
| TwinMapClear(void) | Pesticide | protected |
| TwinMapCopyToMask(void) | Pesticide | protected |
| TwinMapDiffusion(void) | Pesticide | protected |
| TwinMapSpray(LE \*a\_element\_sprayed, double a\_amount) | Pesticide | protected |
| TwinMapSprayCorrectBorders(void) | Pesticide | protected |
| TwinMapSprayPixel(int a\_large\_map\_x, int a\_large\_map\_y, double a\_fractional\_amount) | Pesticide | inlineprotected |
| ~Pesticide(void) | Pesticide | virtual |


- Generated on Thu Jan 10 2013 13:15:36 for ALMaSS Skylark ODdox by
   1.8.1.1
